# An Interpretable Deep-Unfolding Network for Real-Time Simultaneous Multi-Beam Synthesis in Sparse Phased-MIMO Radar

**DOI:** 10.3390/s26206408

**Published:** 2026-10-10

**Authors:** Zhongjia Ma, Zhongmin Pei, Wenyan Wei, Zhangkai Luo, Zengying Yue

**Affiliations:** 1 School of Space Information, Space Engineering University, Beijing 101416, China; mazj25@hgd.edu.cn (Z.M.); wei_wenyan@163.com (W.W.);; 2 National Key Laboratory of Space Target Awareness, Beijing 101416, China

**Keywords:** sparse phased-MIMO radar, simultaneous multi-beam synthesis, deep unfolding, alternating direction method of multipliers, stage-wise penalty sharing, Cholesky factorization reuse, knowledge distillation

## Abstract

Sparse phased multiple-input multiple-output (phased-MIMO) radar combines wide transmit coverage with high-resolution virtual-aperture reception, but simultaneous multi-beam synthesis requires mainlobe shaping, sidelobe control, and interference nulling to be satisfied jointly. Optimization-based solvers preserve these constraints explicitly, yet their long iterative trajectories limit efficient deployment. This paper presents FastUnfoldNet, a physics-structured deep-unfolding framework that maps the alternating direction method of multipliers (ADMM) beamformer onto a fixed-depth architecture. Each layer retains the closed-form sidelobe, null, and mainlobe projections and the standard dual update, while learnable heavy-ball momentum and relaxation reshape the finite-depth trajectory. Stage-wise penalty sharing further couples adaptive optimization with Cholesky factorization reuse, and phase-aligned teacher-response distillation learns a compact 20-layer trajectory for deployment. Simulations covering focused, flat-top, and simultaneous multi-beam synthesis show that the unfolded models reproduce the beam geometry of the parent solver with a substantially shorter update sequence. Depth, ablation, and in-distribution generalization analyses further identify the roles and boundaries of the learned components. FastUnfoldNet therefore provides an interpretable route from constrained beamforming optimization to fixed-depth inference for the considered sparse phased-MIMO setting.

## 1. Introduction

MIMO radar encompasses both colocated configurations, which use waveform diversity to form a virtual aperture, and widely separated configurations, which exploit spatial diversity; standard treatments describe the corresponding signal-processing models and design principles [1,2,3]. Array and waveform design further shape these capabilities: phased-MIMO partitions a colocated aperture into coherently beamformed subarrays, sparse or nested layouts increase spatial degrees of freedom, and probing signals control the transmitted spatial-energy distribution [4,5,6]. In the sparse phased-MIMO setting considered here, the receive beam can be substantially narrower than the illuminated transmit sector; simultaneous multi-beam reception addresses this mismatch by tiling the sector with several high-resolution beams [7].

Each receive beam must achieve three objectives at once: preserve the desired focused or flat-top mainlobe, control the peak sidelobe level (PSL) outside the transition region, and place deep nulls over prescribed interference sectors. The resulting magnitude constraints form a non-convex beam-weight design problem. For a given common region configuration, the beam weights associated with different look directions can be optimized independently [7], but each individual problem remains computationally demanding.

Optimization-based radar design has addressed diversity-based transmit beampattern synthesis, constant-modulus waveforms, and similarity-constrained designs [8,9,10]. Robust and shape-controlled formulations further account for steering-matrix uncertainty, explicit PSL/ISL criteria, and directional energy focusing [11,12,13]. These nonconvex designs are commonly handled through tools such as semidefinite relaxation and ADMM-based variable splitting [14,15]. In the simultaneous multi-beam formulation of [7], ADMM separates the sidelobe, null, and mainlobe constraints into closed-form projections. Its fixed penalties keep the coefficient matrix of the weight update constant, allowing one factorization to be reused across iterations. The remaining cost is the long sequence of matrix-vector products, triangular solves, projections, and dual updates required to approach a high-quality solution.

Deep learning provides a data-driven framework for physical-layer signal processing, while radar-focused surveys summarize its use in colocated MIMO systems [16,17]. Radar applications already include residual-network waveform prediction, interpretable unrolled beampattern design, and data-driven adaptive beamforming [18,19,20]. Algorithm unrolling retains the structure of an iterative solver and traces back to learned sparse-coding iterations [21,22]. Representative architectures include ADMM-Net for compressive-sensing MRI, ISTA-Net for image compressive sensing, and FISTA-Net for imaging inverse problems [23,24,25]. These architectures provide methodological precedents for algorithm unrolling, while their original forward models and objectives differ from those of the constrained radar beam-synthesis problem considered here. Applying them to the present task would require task-specific adaptation of their operators and training objectives. The same model-driven principle has since been applied to MIMO detection, THz hybrid beamforming, and FMCW radar interference mitigation [26,27,28]. Related radar-learning approaches include direct unimodular waveform synthesis and low-complexity joint radar-communication beamforming [29,30]. This structure is especially useful here because the beam-synthesis operators already have clear physical roles.

Unfolding the ADMM beamformer is nevertheless not a direct application of standard unrolling. The most influential trainable quantities are the penalties associated with the three constraint regions, yet these penalties enter the coefficient matrix of the linear weight update. A different penalty at every layer would therefore require a new cubic-cost factorization at every layer, offsetting much of the computational benefit of a short unfolded trajectory.

FastUnfoldNet resolves this penalty–factorization coupling by combining physics-structured ADMM unfolding with stage-wise parameter sharing. The layers retain the original sidelobe truncation, null-disk projection, mainlobe-annulus projection, and standard dual update. The trainable variables comprise the three region penalties, layer-wise relaxation and heavy-ball momentum, and global regularization and null-bound scalars. Penalties are shared within a small number of stages, so each stage uses one cached Cholesky factor, while relaxation and momentum remain layer-dependent.

The contributions are threefold.

(1) We formulate a physics-structured unfolded network for the simultaneous multi-beam ADMM solver of [7]. Each layer exposes the same beamforming states, constraint projections, and standard dual update as the parent iteration, while learnable relaxation and momentum adapt the finite-depth trajectory. This structural inheritance is not presented as a global feasibility or convergence guarantee.

(2) We introduce stage-wise penalty sharing and Cholesky factorization reuse. For *L* layers divided into *P* stages, the number of coefficient-matrix factorizations is reduced from *L* for a naive layer-wise schedule to *P*, under a fixed array, angular grid, problem instance, and shared penalties within each stage.

(3) We compress the 80-layer teacher into a 20-layer student by teacher-strided initialization and phase-aligned response distillation. The depth experiment distinguishes this learned short trajectory from direct truncation of the teacher at the same layer budget.

The experiments evaluate beam quality, convergence with depth, component contributions, computational structure, and in-distribution generalization. The claims are restricted to the tested one-dimensional sparse array and the focused- and flat-top-beam distributions described in Section 4.

The remainder of this article is organized as follows. Section 2 states the signal model, beam-synthesis problem, and parent ADMM iteration. Section 3 presents FastUnfoldNet, its training and distillation, and its structural complexity. Section 4 reports the numerical evidence. Section 5 and Section 6 discuss the scope of the findings and conclude the article.

### Notation

Bold lowercase **a** and bold uppercase **A** denote a vector and a matrix, respectively; *a* denotes a scalar. (⋅)^T^, (⋅)^*^, and (⋅)^H^ denote transpose, conjugate, and conjugate transpose. ℝ*^n^* and ℂ*^n^* are the sets of *n*-dimensional real and complex vectors. $j=\sqrt{-1}$; Re and |⋅| take the real part and the modulus; ∠(⋅) takes the phase. ⊗ is the Kronecker product, diag(**x**) the diagonal matrix formed from **x**, **I** the identity, ∥⋅∥ the Euclidean norm, ∥⋅∥_2_ the spectral norm (largest singular value) of a matrix, ∥⋅∥_∞_ the maximum-modulus (infinity) norm, and ⌊⋅⌋ the floor operator.

## 2. Signal Model, Problem Formulation, and ADMM Foundation

In this section, we first establish the signal model of sparse phased-MIMO radar. Based on this model, we formulate the simultaneous multi-beam synthesis task as constrained beamforming optimization problems. We then develop the ADMM algorithm for solving the problems, which serves as the foundation for the proposed deep-unfolding network.

### 2.1. Signal Model

**Figure 1:**
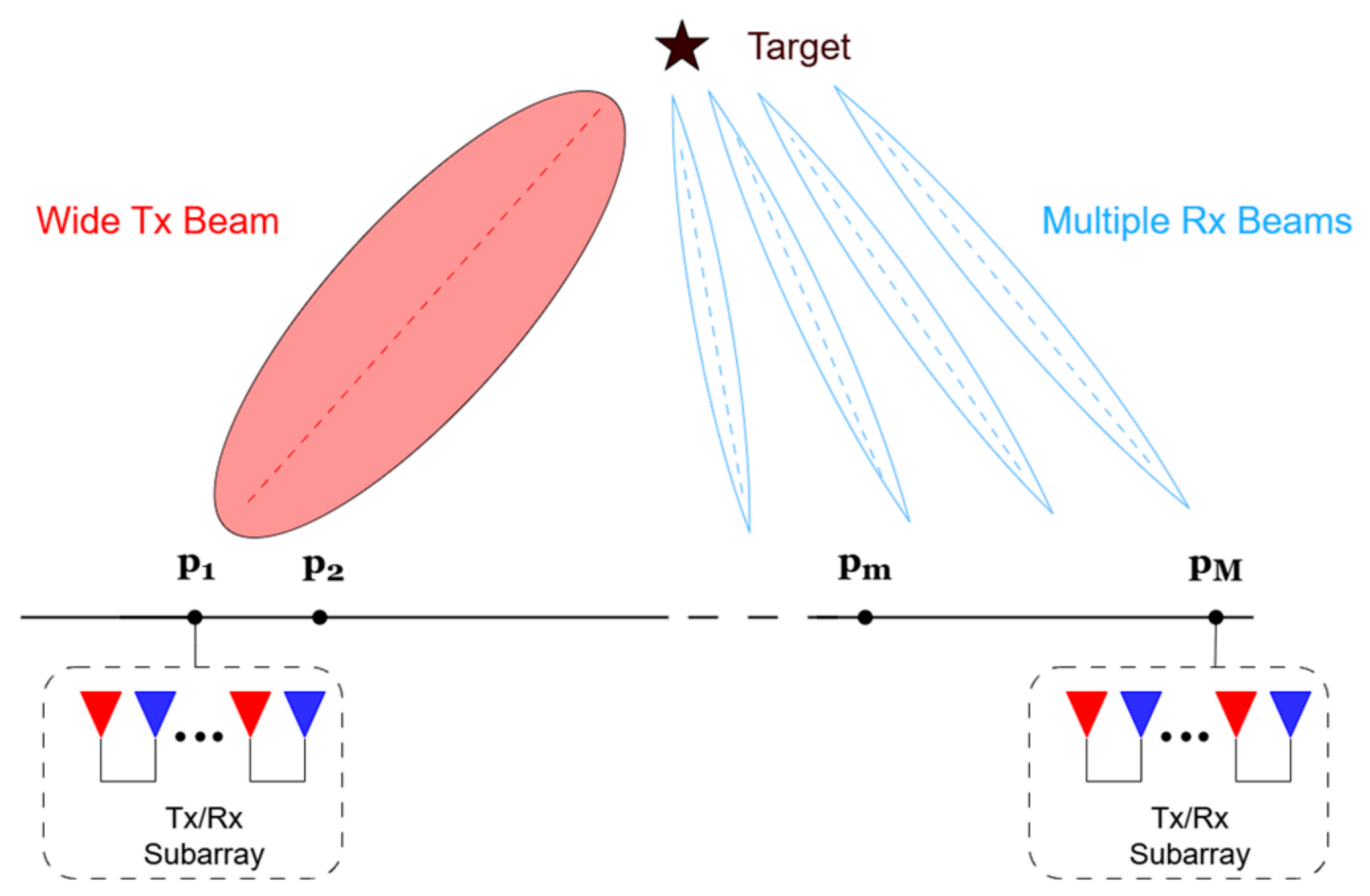
Sparse phased-MIMO radar array. The red ellipse denotes the wide transmit (Tx) beam, the blue narrow beams denote the multiple receive (Rx) beams, and the black star indicates the target. *p*_1_,*p*_2_,…,*p_m_*,…,*p_M_* denote the positions of the Tx/Rx subarrays along the sparse aperture. The red and blue triangular symbols represent the Tx and Rx antenna elements, respectively, and the ellipses indicate omitted intermediate subarrays or antenna elements.

Consider a one-dimensional sparse phased-MIMO radar of *M* colocated subarrays placed at positions **p** = [*p*_1_,…,*p_M_*]^T^ along a large baseline (Figure 1). Each subarray is a uniform linear array of *N* elements spaced by *d* = *λ*/2. The elements within a subarray radiate an identical waveform for coherent gain, whereas different subarrays emit mutually orthogonal waveforms for diversity. Thus, the steering vectors within and between subarrays can be written as:

(1) $$a\left(\theta \right)={\left[1,\;e^{\;j\frac{2\mathrm{\pi d}}{\lambda }\sin\theta },\;\ldots ,\;e^{\;j\frac{2\pi \left(N-1\right)d}{\lambda }\sin\theta }\;\right]}^{T}\in C^{N}$$

and

(2) $$b\left(\theta \right)={\left[\;e^{\;j\frac{2\pi p_{1}}{\lambda }\sin\theta },\;\ldots ,\;e^{\;j\frac{2\pi p_{M}}{\lambda }\sin\theta }\;\right]}^{T}\in C^{M}$$

On transmit, the subarray weights are set by the conventional phase-weighting rule **w***_t_* = **a**(*θ*_t_) that steers to the sector center *θ*_t_, so the transmit beampattern of each subarray is [7]

(3) $$g(\theta )=w_{t}^{H}a(\theta )=a^{H}(\theta_{t})\;a(\theta )$$

All antennas receive the echoes with relative phases determined by their spatial positions. The receive steering vector is therefore the Kronecker product of the across- and within-subarray responses,

(4) $$a_{r}(\theta )=b(\theta )⊗a(\theta )\in C^{\mathrm{MN}}$$

At the receiver, the signals received by the MN antennas are passed through filters matched to the M orthogonal waveforms. Stacking the filter outputs yields the equivalent virtual transmit–receive steering vector,

(5) $$\hat{a}(\theta )=g(\theta )\;\left[b(\theta )⊗a(\theta )\right]⊗b(\theta_{t})\;\in \;C^{M^{2}N}\;$$

With the digital receive-weight vector denoted by $w\in C^{M^{2}N}$, the synthesized beampattern is given by

(6) $$P(\theta )=w^{H}\hat{a}(\theta )$$

The sparse long-baseline aperture provides fine angular resolution and consequently produces a receive mainlobe that is substantially narrower than the wide transmit sector. After matched filtering, the resulting *M*^2^*N* dimensional virtual response is used for digital receive beam synthesis. Therefore, multiple receive beams should be synthesized simultaneously to fully exploit the transmitted energy. Each beam is required to point to a prescribed direction or angular interval while maintaining low sidelobes and deep nulls toward interference directions, which motivates the constrained optimization formulation in the next subsection.

### 2.2. Simultaneous Multi-Beam Optimization and ADMM Baseline

To resolve the width mismatch, *Q* virtual receive beams are synthesized simultaneously from one transmission, each pointing to a different angle within the transmit sector. For *qth* beam with desired direction $\theta_{q}^{⋆}$, the angular axis is partitioned into a mainlobe region $\Omega_{m}^{\left(q\right)}$, which is a single angle for a focused beam or a continuous interval for a flat-top beam; a sidelobe region $\Omega_{s}^{\left(q\right)}$, which lies outside the mainlobe and its surrounding unconstrained transition band; and a null region Ω*_z_*, which contains the interference directions shared by all *Q* beams.

The weight vector **w***_q_* of beam *q* is designed by minimizing its PSL subject to the nulling and mainlobe constraints, where $P_{q}(\theta )=w_{q}^{H}\hat{a}(\theta )$ is the beampattern of *qth* beam,

(7) $$\begin{array}{lll} \underset{w_{q},\;t_{q}}{\min}\; & t_{q} & \\ s.t.\; & \left|P_{q}\left(\theta \right)\right|\leq t_{q}, & \forall \;\theta \in \Omega_{s}^{\left(q\right)},\\ & \left|P_{q}\left(\theta \right)\right|\leq \eta , & \forall \;\theta \in \Omega_{z},\\ & \gamma \leq \left|P_{q}\left(\theta \right)\right|\leq \bar{\gamma }, & \forall \;\theta \in \Omega_{m}^{\left(q\right)}, \end{array}$$

Here *t_q_* is the PSL variable, *η* is the prescribed null bound, and [*γ*,$\bar{\gamma }$] defines the mainlobe ripple. A single angle in $\Omega_{m}^{\left(q\right)}$ gives a focused beam, whereas a continuous interval gives a flat-top beam. The magnitude constraints make each problem non-convex. Given the common angular-region specification, the *Q* weight vectors are optimized independently as in [7].

### 2.3. The Baseline ADMM Solver

A standard way to solve the non-convex problem (7) is the ADMM algorithm [7], which we recall here as the baseline of our work. The beams are independent given the shared null region, so we drop the subscript *q* and present the algorithm for a single beam. Each modulus constraint is decoupled from **w** by an auxiliary variable. Stacking ${\hat{a}}^{H}\left(\theta \right)$ over each region’s angular grid yields the region steering matrices **F**, **G**, **H** (sidelobe, null, mainlobe), which we collectively denote **Φ***_r_* with *r* ∈ *s*,*z*,*m*. Splitting the region responses **Fw**, **Gw**, **Hw** off as auxiliary variables **f**, **g**, **h** recasts problem (7) into the split form

(8) $$\begin{array}{rr} \underset{w,t,f,g,h}{\min}\; & t\;\\ s.t.\; & \left\{\begin{array}{ll} f=\mathrm{Fw}, & |f_{s}|\leq t,\\ g=\mathrm{Gw}, & |g_{z}|\leq \eta ,\\ h=\mathrm{Hw}, & \gamma \leq |h_{m}|\leq \bar{\bar{\gamma }}, \end{array}\right. \end{array}$$

where *s*, *z*, and *m* index the sidelobe, null, and mainlobe samples, respectively. With fixed penalties *ρ_r_* > 0 and unscaled dual variables **λ***_r_*, the augmented Lagrangian of Equation (8) is

(9) $$L=t+\sum_{r}\frac{\rho_{r}}{2}{∥z_{r}-\Phi_{r}w+\lambda_{r}/\rho_{r}∥}_{2}^{2},\;\;z_{r}\in \{f,g,h\},$$

with the constant multiplier terms omitted; the modulus constraints in Equation (8) are enforced in the **z***_r_*-updates by the Euclidean projections (11)–(12). ADMM minimizes (9) over one block at a time, which yields four structured subproblem updates per iteration; we state the resulting updates here and refer to [7] for the derivations.

(1) Weight update. Minimizing (9) over **w**, with a small regularizer *ϵ***I** ensuring invertibility, is an unconstrained quadratic problem, so **w**^(k+1)^ solves the linear system
  (10) $$\begin{array}{ll} Q\;w^{\left(k+1\right)} & =r^{\left(k\right)},\;\\ Q & =\epsilon I+\sum_{r\in \{s,z,m\}}\rho_{r}\Phi_{r}^{H}\Phi_{r},\\ r^{\left(k\right)} & =\sum_{r\in \left\{s,z,m\right\}}\rho_{r}\;\Phi_{r}^{H}\left(z_{r}^{\left(k\right)}+\frac{\lambda_{r}^{\left(k\right)}}{\rho_{r}}\right). \end{array}$$
(2) Sidelobe update. Define $f=Fw^{\left(k+1\right)}-\lambda_{s}^{\left(k\right)}/\rho_{s}$. The PSL *t*^(*k*+1)^ that minimizes *t* subject to |*f_s_*| ≤ *t* is obtained by a bisection search [7], after which each sidelobe auxiliary is truncated onto the disk of radius *t*^(*k*+1)^,
  (11) $$f_{s}^{\left(k+1\right)}=\left\{\begin{array}{ll} {\tilde{f}}_{s}, & |{\tilde{f}}_{s}|\leq t^{\left(k+1\right)},\\ t^{\left(k+1\right)}\;{\tilde{f}}_{s}/|{\tilde{f}}_{s}|, & |{\tilde{f}}_{s}|>t^{\left(k+1\right)}. \end{array}\right.$$
(3) Null and mainlobe updates. With $g=Gw^{\left(k+1\right)}-\lambda_{z}^{\left(k\right)}/\rho_{z}$ and $h=Hw^{\left(k+1\right)}-\lambda_{m}^{\left(k\right)}/\rho_{m}$, each null response is projected onto the disk of radius *η* and each mainlobe response onto the annulus [*γ*,$\bar{\gamma }$],
  (12) $$g_{z}^{\left(k+1\right)}=\left\{\begin{array}{ll} g_{z}, & |g_{z}|\leq \eta ,\\ \eta \;g_{z}/|g_{z}|, & |g_{z}|>\eta , \end{array}\right.\;\;h_{m}^{\left(k+1\right)}=\left\{\begin{array}{ll} \gamma \;h_{m}/|h_{m}|, & |h_{m}|<\gamma ,\\ \bar{\gamma }\;h_{m}/|h_{m}|, & |h_{m}|>\bar{\gamma },\\ h_{m}, & \mathrm{otherwise}. \end{array}\right.$$
(4) Dual update. A dual ascent with step ρ_r_ closes the iteration,
  (13) $$\lambda_{r}^{\left(k+1\right)}=\lambda_{r}^{\left(k\right)}+\rho_{r}\left(z_{r}^{\left(k+1\right)}-\Phi_{r}w^{\left(k+1\right)}\right),\;\;r\in \{s,z,m\}.$$

Updates (10)–(13) are iterated until the primal residuals fall below *ϵ*_tol_ or the budget *K*_max_ is reached.

Because the penalties are fixed, ***Q*** is constant throughout Algorithm 1; its Cholesky factorization can be computed once and reused in every iteration. The factorization is therefore amortized, but the solver still executes *K*_(ADMM)_ repeated products, triangular solves, projections, and dual updates. This repeated trajectory, rather than the inherited formulation itself, is the bottleneck addressed by FastUnfoldNet.
**Algorithm 1** Baseline ADMM beamformer for a single beamRequires: Region matrices and bounds; fixed penalties *ρ_r_*; tolerance *ϵ*_(tol)_; budget *K*_(max)_ 1: Assemble Q by Equation (10) and compute its Cholesky factor once 2: Initialize w, auxiliary variables, and dual variables 3: Repeat until the residual tolerance or iteration budget is reached 4: Update w by the cached linear solve (10) 5: Project the sidelobe responses by Equation (11) 6: Project the null and mainlobe responses by Equation (12) 7: Apply the standard dual update (13) Ensure: Beam weight w

## 3. The Proposed FastUnfoldNet

**Figure 2:**
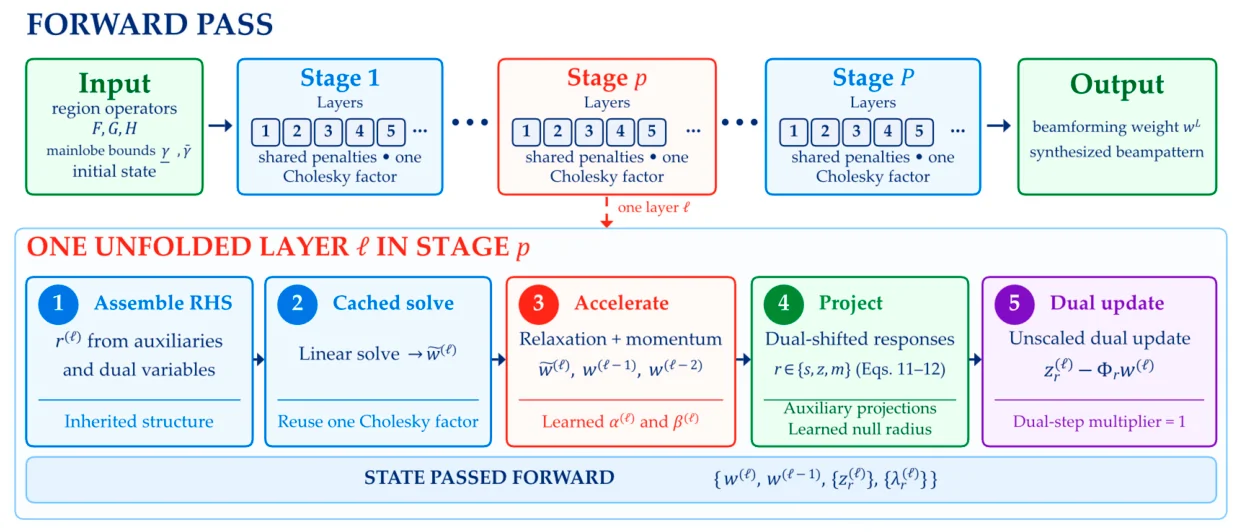
FastUnfoldNet architecture. (**Top**): the unrolled forward pass through *P* stages. (**Bottom**): one layer runs as five differentiable operators derived from a single ADMM iteration. Arrows indicate the forward flow of states and information. Colors are used to visually distinguish the functional blocks. The numbered circles 1–5 indicate the sequence of the five operators within one unfolded layer. Ellipses within the stage blocks denote omitted intermediate layers, whereas ellipses between stage blocks denote omitted intermediate stages. Mathematical symbols are defined in the main text.

FastUnfoldNet unfolds the computational structure of *L* ADMM iterations into *L* feedforward layers. The projection operators and the standard dual update are inherited directly from Algorithm 1; stage-wise penalties, layer-wise relaxation, and layer-wise momentum control the learned trajectory. Section 3.1, Section 3.2 and Section 3.3 describe the layer and factorization reuse, Section 3.4 and Section 3.5 present network training and teacher-response guidance, and Section 3.6 states the resulting structural complexity. Figure 2 provides an overview of the stage-wise unfolded architecture and the five operators executed in each layer.

### 3.1. Unrolled Architecture and Inherited Operators

Layer *l* receives the state $\left(w^{\left(l-1\right)},w^{\left(l-2\right)},\{z_{r}^{\left(l-1\right)}\},\{\lambda_{r}^{\left(l-1\right)}\}\right)$, with *r* ∈ *s*,*z*,*m* indexing the sidelobe, null, and mainlobe regions, and transforms it through the five operators listed in Algorithm 2 and sketched in Figure 2. The primal output from layer *l* − 2 is retained only as the one-step history required by the heavy-ball term in Equation (16); no other interlayer memory is used. Four of the five operators are inherited from Algorithm 1 and were stated in Equations (10)–(13); the additional momentum-and-relaxation operator is the learnable acceleration deferred to Section 3.2.
**Algorithm 2** One unrolled layer of FastUnfoldNet (layer *l*)Requires: Previous state; cached factor of stage *s*(*l*); learned *α*^(*l*)^, *β*^(*l*)^, and stage penalties 1: Assemble the right-hand side of Equation (10) from the previous auxiliary and dual variables 2: Compute the raw weight update with the cached factor, as in Equation (14) 3: Apply the relaxation and heavy-ball momentum update (16) 4: Apply the sidelobe, null, and mainlobe projections (11)–(12) 5: Apply the standard ADMM dual update (15) Ensure: Updated beamforming state

Step 1 assembles the same right-hand side as the baseline weight update (10), evaluated with the auxiliaries and duals carried over from the previous layer; its cost is *O*(*TD*), with *T* the total grid size and *D* the virtual dimension.

The normal equations are solved with the Cholesky factor of the stage to which layer *l* belongs,

(14) $${\tilde{w}}^{\left(l\right)}=Q_{s(l)}^{-1}\;r^{\left(l\right)},$$

where *s*(*l*) = ⌊(*l* − 1)*P*/*L*⌋+1 is the stage index defined in Section 3.3. Because **Q***_s_* is factorized once on entry to its stage, this step performs an O (*D*^2^) triangular back-substitution rather than a fresh O(*D*^3^) factorization.

The raw solve ${\tilde{w}}^{\left(l\right)}$ is not passed to the projections directly; it is first accelerated by the learnable momentum and relaxation update of Section 3.2.

The accelerated weights are then used to form the three dual-shifted region responses, which are mapped onto their respective constraint sets by the projections (11)–(12) of Algorithm 1. Each region projects its dual-shifted response: sidelobe samples are truncated at the bisection-derived level, null samples are clipped to the prescribed disk, and mainlobe samples are projected onto the admissible annulus. These tensor operations are differentiable almost everywhere and are trained using automatic differentiation with the implementation’s defined subgradients at boundary points; no straight-through estimator is introduced. The projected auxiliary responses satisfy their respective sets by construction, whereas exact feasibility of the finite-depth weight output also depends on primal consistency. Accordingly, we do not claim global feasibility or convergence of the learned finite-depth dynamics.

The dual variables then follow the standard unscaled ADMM update for each constraint region,

(15) $$\lambda_{r}^{\left(l\right)}=\lambda_{r}^{\left(l-1\right)}+\rho_{r}^{\left(s(l)\right)}(z_{r}^{\left(l\right)}-\Phi_{r}w^{\left(l\right)})$$

where $\rho_{r}^{\left(s\left(l\right)\right)}$ is the stage-wise region penalty associated with layer *l*. No additional dual-step multiplier is learned. The updated weights, auxiliary responses, and dual variables are passed to layer *l* + 1.

### 3.2. Learnable Heavy-Ball Momentum and Relaxation

A short unfolded network must make useful progress within a prescribed layer budget. FastUnfoldNet therefore applies a learnable heavy-ball-inspired inertial correction [31] to the raw solve of Equation (14), followed by relaxation with the previous layer output,

(16) $$w^{\left(l\right)}=(1-\alpha^{\left(l\right)})\;w^{\left(l-1\right)}+\alpha^{\left(l\right)}\left[{\tilde{w}}^{\left(l\right)}+\beta^{\left(l\right)}\left({\tilde{w}}^{\left(l\right)}-w^{\left(l-2\right)}\right)\right].$$

The momentum coefficient *β*^(l)^ introduces an inertial extrapolation based on the difference between the current raw solve ${\tilde{w}}^{\left(l\right)}$ and the two-step-back primal state $w^{\left(l-2\right)}$. Learning it per layer lets the early and late parts of the trajectory use different amounts of inertia, while the bounded parameterization limits excessive extrapolation.

The relaxation coefficient $\alpha^{\left(l\right)}$ blends the accelerated candidate with the previous iterate; values below or above unity correspond to under-relaxation or over-relaxation of this candidate, respectively. The neutral values *α*^(*l*)^ = 1 and *β*^(*l*)^ = 0 recover the unaccelerated primal weight update, so FastUnfoldNet contains the corresponding neutral-acceleration configuration as a controlled ablation.

The acceleration acts before the inherited projections and does not replace their feasible-set geometry. This is a structural statement about the layer, not a claim of global convergence for the learned finite-depth dynamics; their effect is evaluated through the trajectory and component ablations in Section 4.

### 3.3. Stage-Wise Penalty Sharing and Cholesky Caching

The three region penalties are natural candidates for learning because they control the balance among the sidelobe, null, and mainlobe subproblems. They also enter the coefficient matrix. If every layer used a distinct penalty triplet, the matrix

(17) $$Q^{\left(l\right)}=\left(\rho_{s}^{\left(l\right)}F^{H}F+\rho_{z}^{\left(l\right)}G^{H}G+\rho_{m}^{\left(l\right)}H^{H}H\right)+\epsilon I$$

would change at every layer, leading to *L* Cholesky factorizations with cubic dependence on *D*.

We avoid this by sharing penalties within contiguous stages. The *L* layers are partitioned into *P* stages by

(18) $$s(l)=\left\lfloor \frac{(l-1)P}{L}\right\rfloor +1,\;l=1,\ldots ,L$$

Thus, the stage matrix $Q_{s(l)}$ is constant for all layers within the same stage. For a fixed problem instance, array, angular grid, and stage penalty triplet, one Cholesky factor is computed on entry to a stage and reused by its layers. The solve in Equation (14) then uses triangular substitutions rather than a new factorization. Accordingly, the factorization-and-solve component changes from

(19) $$O(LD^{3})\;\to \;O(PD^{3}+LD^{2}).$$

This changes the factorization term from one per layer to one per stage. With *L* = 80 and *P* = 4, the teacher uses four factorizations rather than 80 for a naive layer-wise penalty schedule. The regularization in **Q** makes each stage matrix Hermitian positive definite, so the cached Cholesky factor is well defined [32].

Stage-wise sharing therefore preserves schedule flexibility at a coarser resolution while restoring factorization reuse. It does not reduce the *P* stage factorizations to the single factorization of fixed-penalty ADMM; the exact complexity comparison is given in Section 3.6.

### 3.4. Training Objective and Deep Supervision

FastUnfoldNet learns only 174 scalars in the 80-layer teacher and 54 in the 20-layer student. Table 1 groups them by scope; every scalar controls a defined operation in the unfolded solver.

#### 3.4.1. Parameterization of the Learnable Scalars

FastUnfoldNet has no conventional hidden-layer activation such as ReLU. Its nonlinear activation functions instead map unconstrained raw scalars to admissible algorithmic ranges before those scalars enter the unfolded updates. The sigmoid and hyperbolic tangent mappings place relaxation and momentum at their neutral values when the raw variables are zero; exponential mappings enforce positive penalties and regularization; and explicit clipping bounds the mapped penalties, regularization, and null-bound quantities to numerically meaningful intervals.

1. Relaxation: *α* = 1/2 + *σ*(*ϑ_α_*) ∈ (0.5,1.5); *σ*(*x*) = 1/(1 + *e*^(−*x*)^) and *α* = 1 at *ϑ_α_* = 0.
2. Momentum: *β* = 0.6tanh(*ϑ_β_*) ∈ (−0.6,0.6), with *β* = 0 at *ϑβ* = 0.
3. Region penalties: *ρ_r_* = clamp(exp(*ϑ*_(*ρ*,*r*)_),10^(−3)^,50), *r* ∈ *s*,*z*,*m*, which enforces positivity and supports log-scale learning.
4. The implementation parameterizes *ϵ*_reg_ = clamp(exp(*ϑ_ϵ_*),10^−4^,0.1), with the regularization coefficient in Equation (10) given by *ϵ* = 2*ϵ*_reg_ under the equivalent half-scaled linear system used for the Cholesky solve. The null bound is represented in decibels as *η*_(dB)_ = clamp(*ϑ_η_*,−80,−30) and converted to amplitude by $\eta =10^{\left(\eta_{\left(\mathrm{dB}\right)}/20\right)}$. The dual ascent multiplier is fixed to unity and is not included in the learnable parameter set.

These mappings act as constrained parameterizations rather than feature-extraction activations. Consequently, every learned scalar remains associated with an identifiable operation in the parent solver.

#### 3.4.2. Physical Loss and Deep Supervision

The teacher is trained end-to-end with beam-domain deep supervision. At each layer, the mainlobe-window term is evaluated in the linear power domain, the null and sidelobe terms are evaluated in decibels relative to the current-layer mainlobe peak, and the weight-normalization term is evaluated directly from the beamforming-weight norm. The physical loss is evaluated at every layer and combined with weights proportional to log(1 + *l*), normalized by their sum as in Equation (20), so later layers receive greater emphasis while gradients still reach the full trajectory. The teacher is not forced to reproduce the intermediate iterates of ADMM.

(20) $$L=\frac{1}{\sum_{l=1}^{L}\log\left(1+l\right)}\sum_{l=1}^{L}\log\left(1+l\right)\;L_{\mathrm{phys}}\left(w^{\left(l\right)}\right).$$

The per-layer physical objective combines six weighted components: a mainlobe-window term, a worst-null hinge, a top-*K* PSL hinge, a grid-wide sidelobe hinge, a sidelobe-uniformity term, and a weight-normalization term. Their coefficients are held fixed within each training configuration.

1. Mainlobe-window loss *L*_(main)_ applies hinge penalties only where the focused or flat-top response falls below the prescribed lower bound or exceeds the upper ripple bound.

2. Null loss is the positive excess of the worst mainlobe-normalized null response over the sample-specific target. The target is defined as $\tau_{\mathrm{null}}^{\left(i\right)}=\max\left(\eta_{\mathrm{cur}},d_{\mathrm{ADMM}}^{\left(i\right)}-m\right)$, where $d_{\mathrm{ADMM}}^{\left(i\right)}$ is the null depth of the corresponding ADMM training reference and *m* is the configured margin. This prevents the curriculum from requiring a null target more than *m* dB deeper than the ADMM reference when that target would otherwise be overly stringent.

3. PSL loss *L*_psl_ applies a hinge to the *K* strongest sidelobe samples, whereas the grid-wide term *L*_all_ penalizes every sidelobe sample above the same target. Their combination controls both isolated peaks and a raised sidelobe floor.

4. Sidelobe-uniformity loss *L*_uni_ is the variance of the active sidelobe envelope in decibels, and *L*_norm_ penalizes deviation of the beamforming-weight norm from unity. Together, these terms regularize residual sidelobe energy and the scale of the solution.

#### 3.4.3. Training Procedure

The scalars are optimized with Adam using prescribed null-depth and PSL target schedules. Algorithm 3 summarizes the procedure, and Section 4.1 reports the data and optimization settings.

(21) $$\mathrm{score}=\left\{\begin{array}{cc} \max\left(\Delta \mathrm{PSL},\Delta \mathrm{Null}\right), & \mathrm{focused}\;\mathrm{beam},\\ \max\left(\Delta \mathrm{PSL},\Delta \mathrm{Null},\Delta \mathrm{Ripple}\right), & \mathrm{flat}-\mathrm{top}\;\mathrm{beam}. \end{array}\right.$$

Here, each gap is defined as the network metric minus the corresponding ADMM-reference metric; positive values are worse and negative values are better. For flat-top training, ΔRipple is additionally included to account for mainlobe ripple.
**Algorithm 3** Training procedure of FastUnfoldNet**Requires:** training set generated by ADMM; depth *L*; stages *P*; curriculum targets 1: Initialize *α* and *β* at their neutral values and initialize penalties, *ϵ*, and *η* at their prescribed solver values 2: For each target schedule, do 3: Set the null-depth and PSL targets prescribed for the current stage 4: For each epoch: run Algorithm 2 for *l* = 1,…,*L*, evaluate the deep-supervised loss (20), update the scalars by Adam 5: End for 6: Retain the checkpoint with the lowest validation score (21)
**Ensure:** Training schedule $\left\{\alpha^{\left(l\right)},\;\beta^{\left(l\right)},\;\rho_{r}^{\left[s\right]},\;\eta \right\}$


### 3.5. Student Training and Teacher-Response Guidance

Following the teacher–student knowledge-distillation paradigm [33], the 80-layer teacher is compressed into a 20-layer student. Directly truncating the teacher exposes an intermediate state rather than a complete short-depth solution, so the student is optimized as a distinct trajectory against the frozen teacher’s final beamforming response. The base student objective combines the beam-domain specification loss with a phase-aligned final-response guidance term from the frozen teacher.

(22) $$L_{\mathrm{student}}^{\mathrm{base}}=L_{\mathrm{spec}}+\lambda_{D}L_{\mathrm{PA}},\lambda_{D}=0.5$$

Here, ℒ_spec_ is the deep-supervised sum of mainlobe-window, null, top-K PSL, grid-wide sidelobe, sidelobe-uniformity, and weight-normalization losses. The respective fixed weights are (0.20, 0.25, 0.30, 0.08, 0.15, 0.10) for focused and (0.25, 0.25, 0.30, 0.08, 0.15, 0.10) for flat-top students; they are not claimed to be optimal. For final weights **w***_S_* and **w***_T_* of the student and frozen teacher, set $c=(w_{T}^{H}w_{S})/(|w_{T}^{H}w_{S}|+\delta )$ and $L_{\mathrm{PA}}=D^{-1}{\left\|c^{*}w_{S}-w_{T}\right\|}_{2}^{2}$ (δ = 10^−12^ for numerical stability). Only final responses are matched; intermediate teacher states are not.

The student is initialized by copying *α* and *β* from every fourth teacher layer and copying all stage-wise and global scalars. It is then trained end-to-end from this initialization using the base objective in (22). This initialization transfers the coarse shape of the teacher schedule while allowing the 20-layer student to learn a distinct trajectory.

The teacher is used only during offline initialization and training. Once training is complete, the student performs its 20-layer inference using its own learned parameters, without requiring the teacher network or its outputs.

### 3.6. Computational Complexity and Structural Properties

Let *T* denote the total number of angular samples used by the three region operators. For a single problem instance, constructing the region Gram matrices requires *O*(*D*^2^*T*) operations. Given these matrices, FastUnfoldNet with *L* unfolded layers and *P* stages requires *O*(*PD*^3^ + *L*(*DT* + *D*^2^)) operations: one *O*(*D*^3^) Cholesky factorization per stage, followed in each layer by *O*(*DT*) steering products and response evaluation, a *O*(*D*^2^) cached linear solve, and *O*(*T*) projections and dual updates. Thus, including the common matrix-construction cost, the overall complexity is *O*(*D*^2^*T* + *PD*^3^ + *L*(*DT* + *D*^2^)). The fixed-penalty parent ADMM has the corresponding complexity *O*(*D*^2^*T* + *D*^3^ + *K*_ADMM_(*DT* + *D*^2^)), where *K*_ADMM_ denotes the number of iterations actually executed. The fixed bisection counts used in the sidelobe projection affect constant factors but not these asymptotic orders.

Three structural properties follow. First, the regularized stage matrices are Hermitian positive definite, so their Cholesky factors exist. Second, with *α* = 1 and *β* = 0, and with the region penalties and global parameters fixed at their parent values, the unfolded layer recovers the algebraic form of the corresponding parent primal update, constraint projections, and standard dual ascent. Third, stage-wise sharing changes the number of factorizations from *L* to *P* under the caching conditions stated in Section 3.3. These properties do not constitute a global convergence guarantee for the learned finite-depth dynamics. Additional structural properties of the proposed unfolded architecture are provided in Appendix A.

## 4. Numerical Simulations

We evaluate whether FastUnfoldNet approximates the parent ADMM solver within a fixed layer budget and identify which design choices support that approximation. The evidence proceeds from beam quality to convergence and depth, component ablation, computational structure, and generalization over random focused-beam configurations. ADMM denotes Algorithm 1 run until the residual tolerance is met, with 1000 iterations as the maximum; FUN-80L and FUN-20L denote the teacher and distilled student, respectively.

### 4.1. Experimental Setup and Evaluation Protocol

The array follows [7] with *M* = 6 subarrays, *N* = 6 elements per subarray, a 1 GHz carrier (*λ* = 0.3 m), subarray positions [0, 1.60, 3.04, 4.52, 6.97, 9.25] m, and a transmit steering direction of 20°, giving a virtual dimension of *D* = *M*^2^*N* = 216. The angular grid spans [−90°, 90°] with 0.1° spacing, resulting in 1801 angular samples. Table 2 lists the remaining settings.

All methods are evaluated using the same array configuration and beam-synthesis specifications to provide a common basis for comparison. The focused, flat-top, and simultaneous multi-beam scenarios assess different synthesis requirements within this configuration; they do not establish generalization across array types.

#### Training and Metrics

Focused and flat-top models are trained on separately generated training and validation sets with a batch size of 8. The detailed beam-type-specific dataset configurations are summarized in Table 2. The six multi-beam test scenarios summarized in Table 3, including the two representative cases visualized later, together with the 1000-configuration sweep, are used exclusively for evaluation rather than checkpoint selection. Training uses Adam optimization, cosine learning-rate annealing, and gradient clipping. The target schedule is configuration-dependent: the focused student progressively tightens its null and PSL targets during training, whereas the final teacher configurations and the flat-top student use fixed target schedules.

PSL is defined as the maximum normalized response over the sidelobe region outside the mainlobe, transition region, and prescribed null regions, whereas null depth is determined by the maximum normalized response within the prescribed null regions; more negative values indicate better suppression. Every reported gap is defined as network minus ADMM, so a positive gap indicates worse performance than ADMM, whereas a negative gap indicates better performance. The −70 dB training target and the −60 dB threshold used in the generalization experiment serve different purposes and should not be interpreted interchangeably.

The multi-beam geometries reproduce the six scenarios of [7]. The networks use distribution-level training, so the comparison evaluates finite-depth acceleration over the tested beam-synthesis scenarios rather than universal superiority over an optimizer.

### 4.2. Beam Synthesis Quality

**Figure 3:**
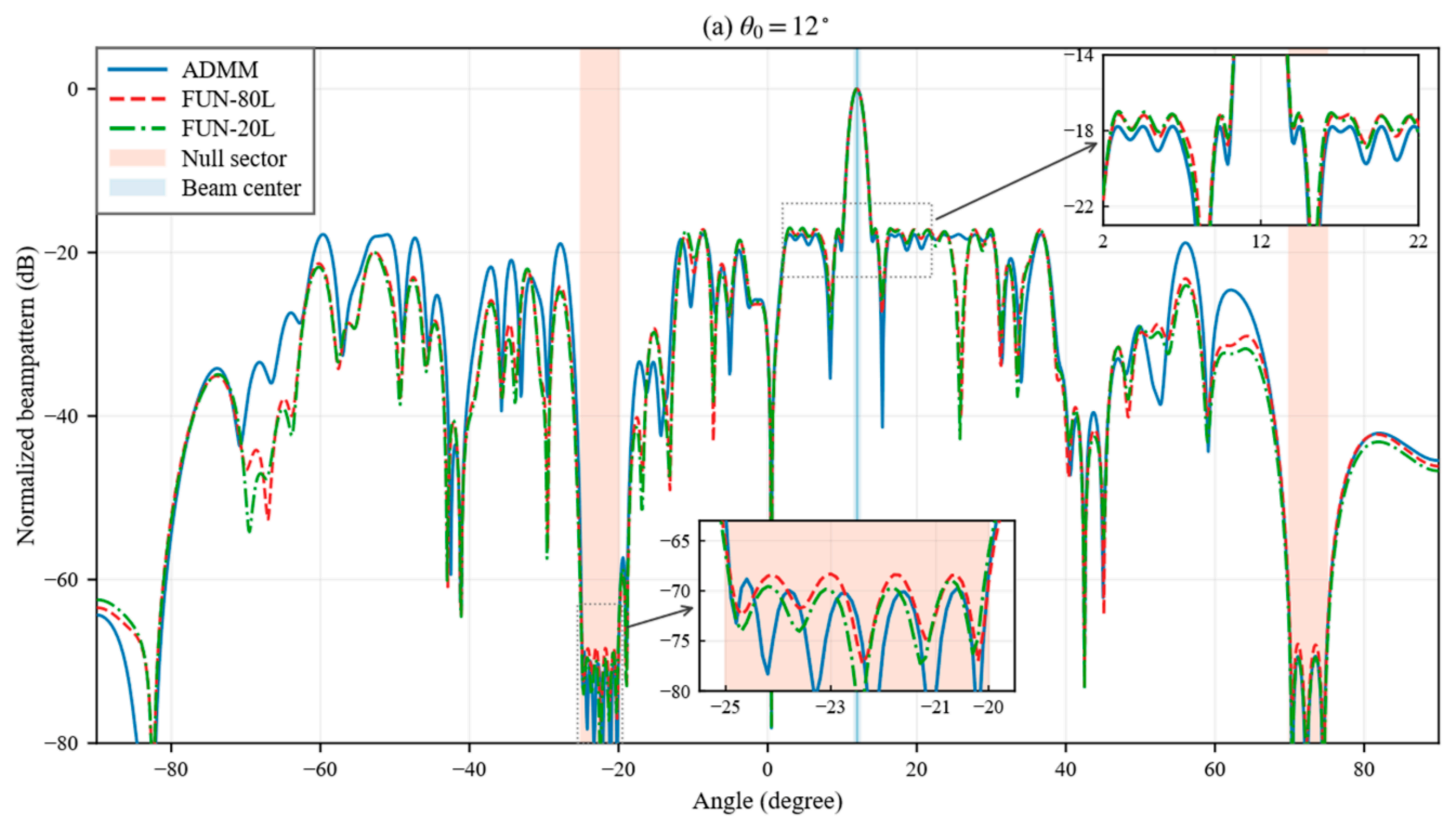

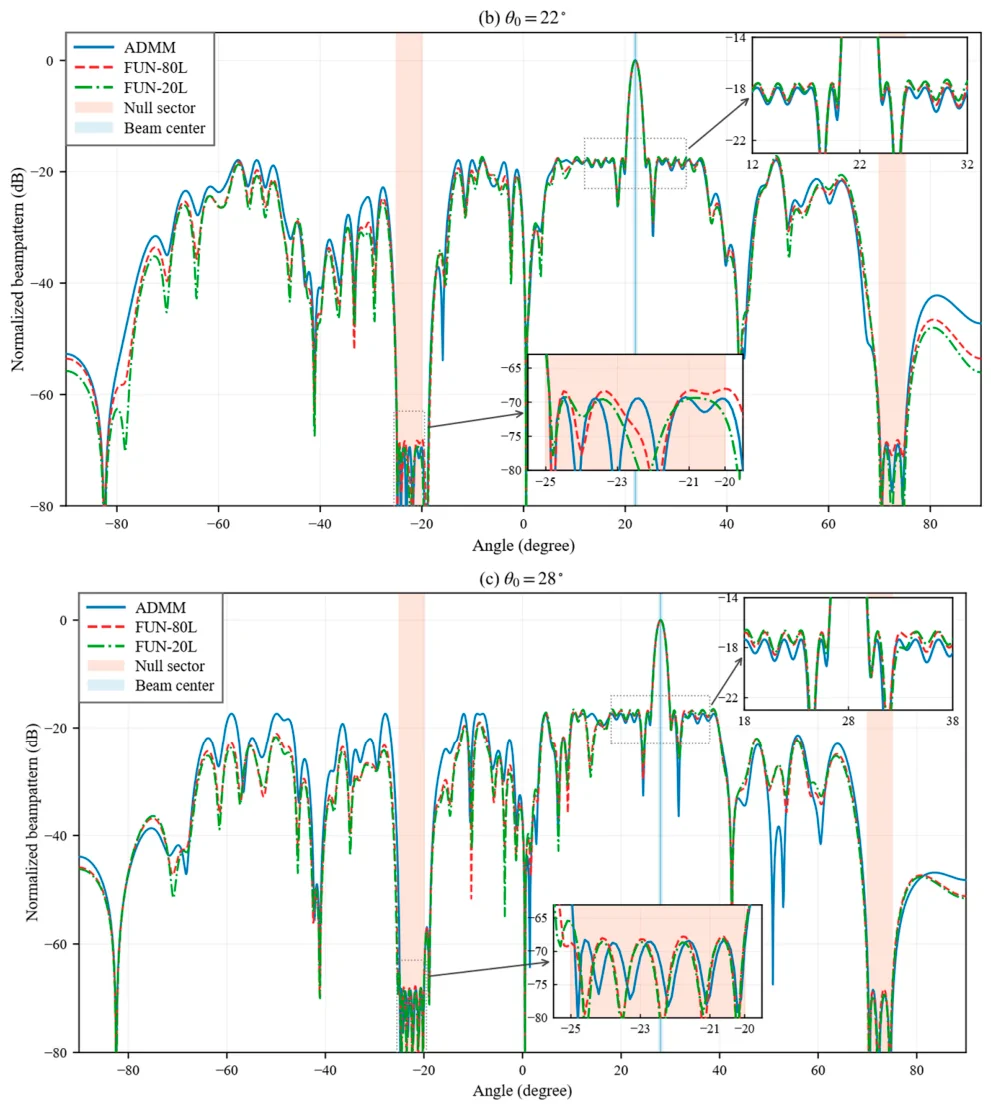
Representative focused-beam synthesis for three look directions: (**a**) 12°, (**b**) 22°, and (**c**) 28°. Each panel compares ADMM, FUN-80L, and FUN-20L.

**Figure 4:**
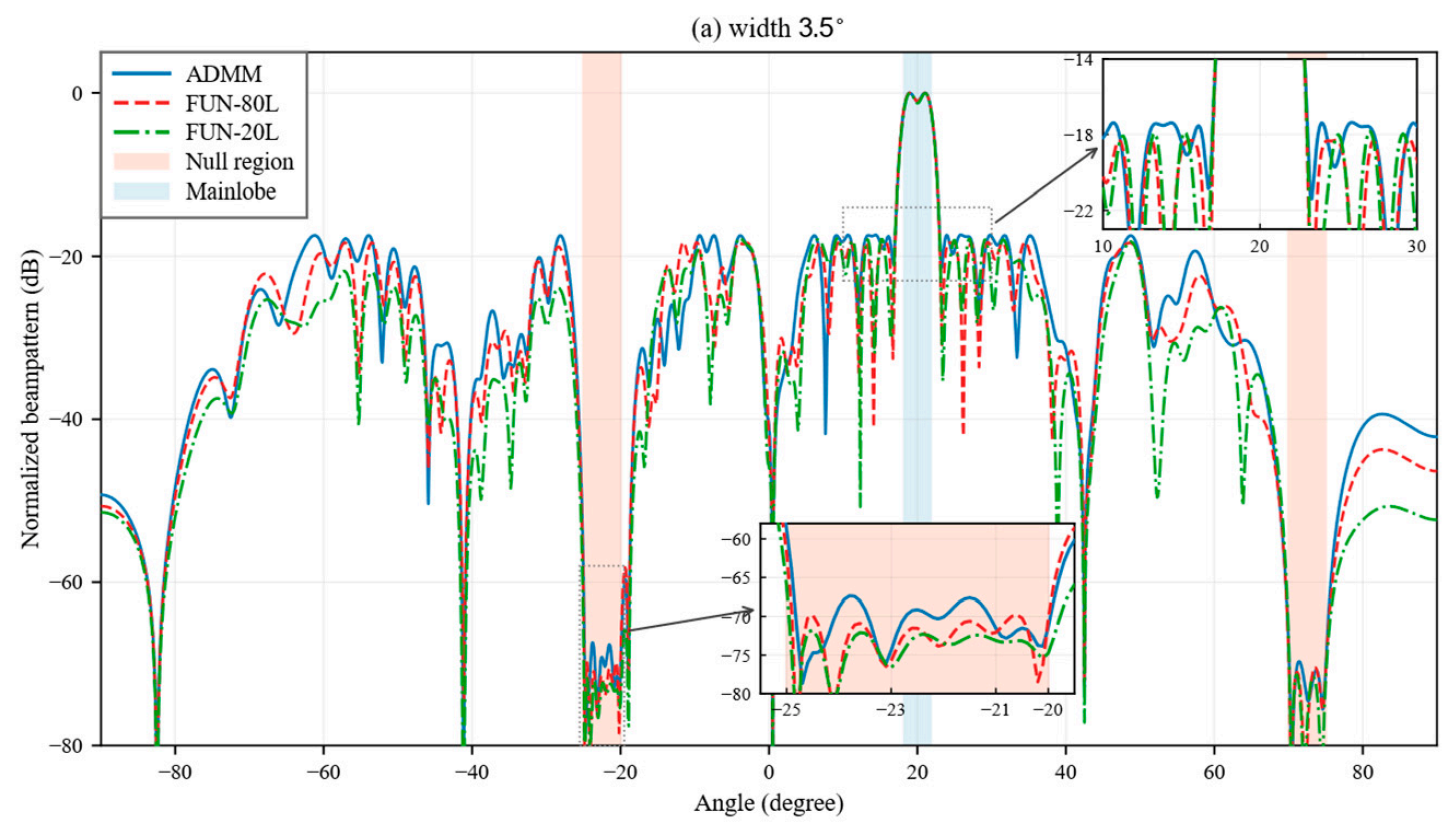

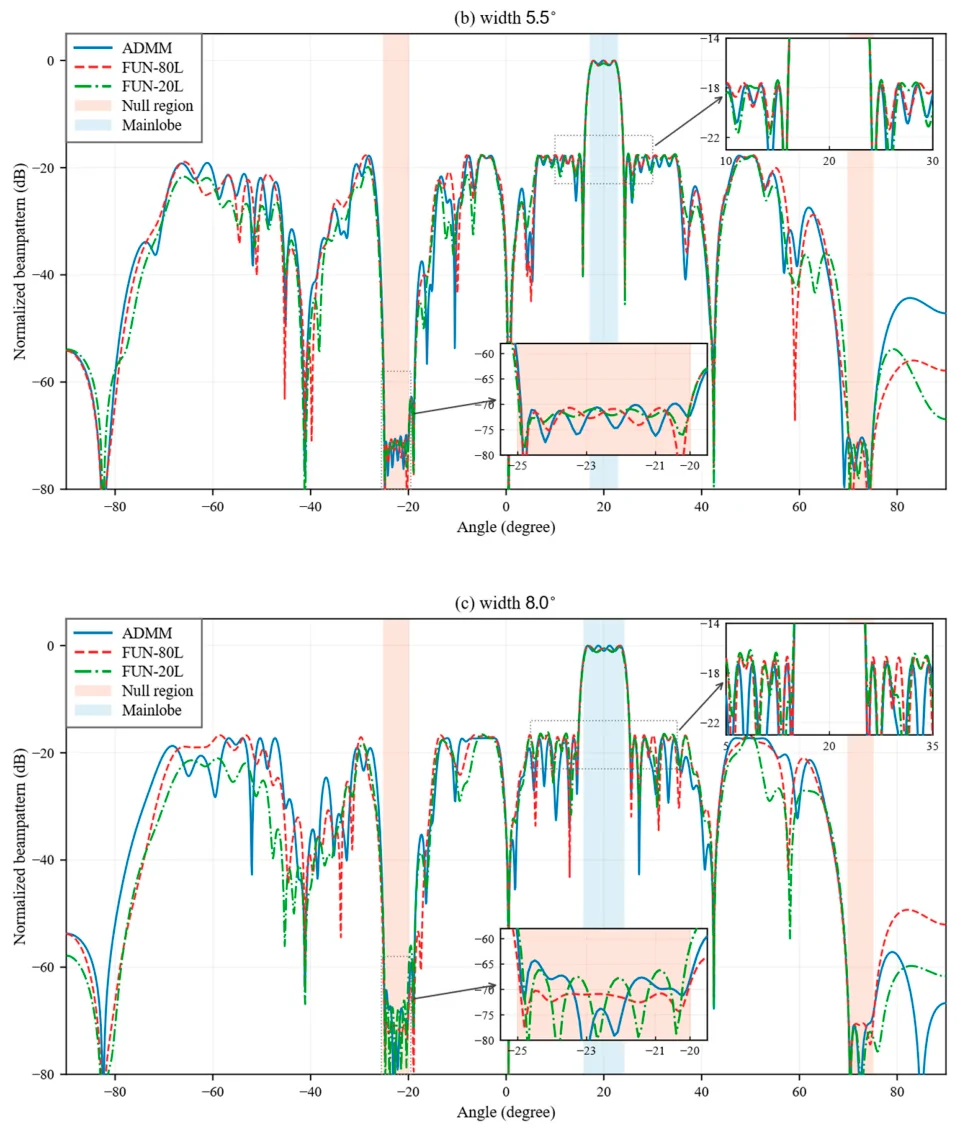
Representative flat-top-beam synthesis for three mainlobe widths: (**a**) 3.5°, (**b**) 5.5°, and (**c**) 8.0°. Each panel compares ADMM, FUN-80L, and FUN-20L.

We first test whether the shortened trajectories approximate the focused and flat-top solutions of ADMM. Figure 3 and Figure 4 show representative focused- and flat-top-beampatterns, respectively. The unfolded models retain the intended coverage geometry and place the prescribed null regions, although small mainlobe-ripple deviations remain in some flat-top cases. The principal differences occur in the sidelobe peaks, worst null samples, and finite-depth mainlobe fidelity; the results therefore support close approximation rather than exact constraint satisfaction.

**Figure 5:**
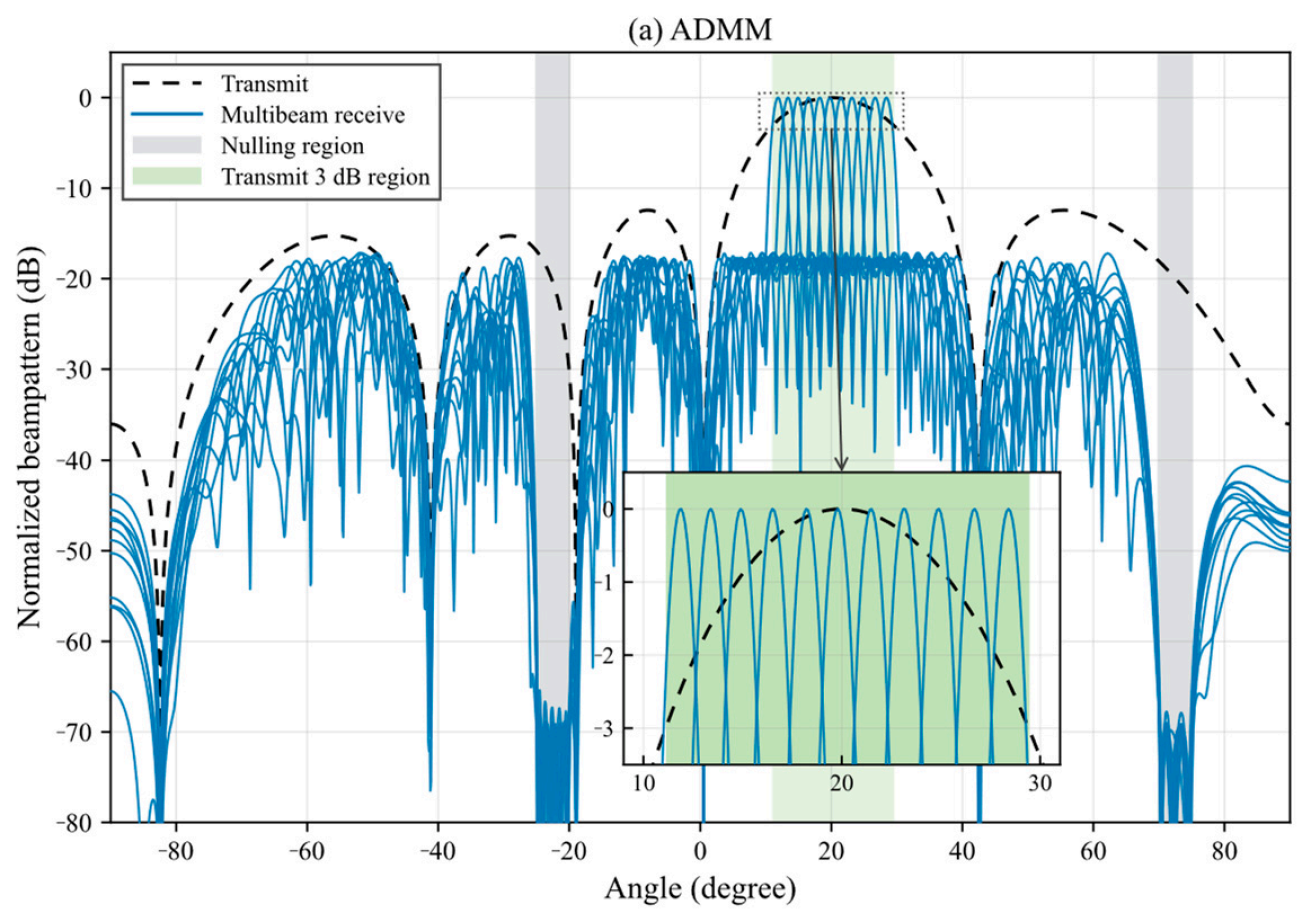

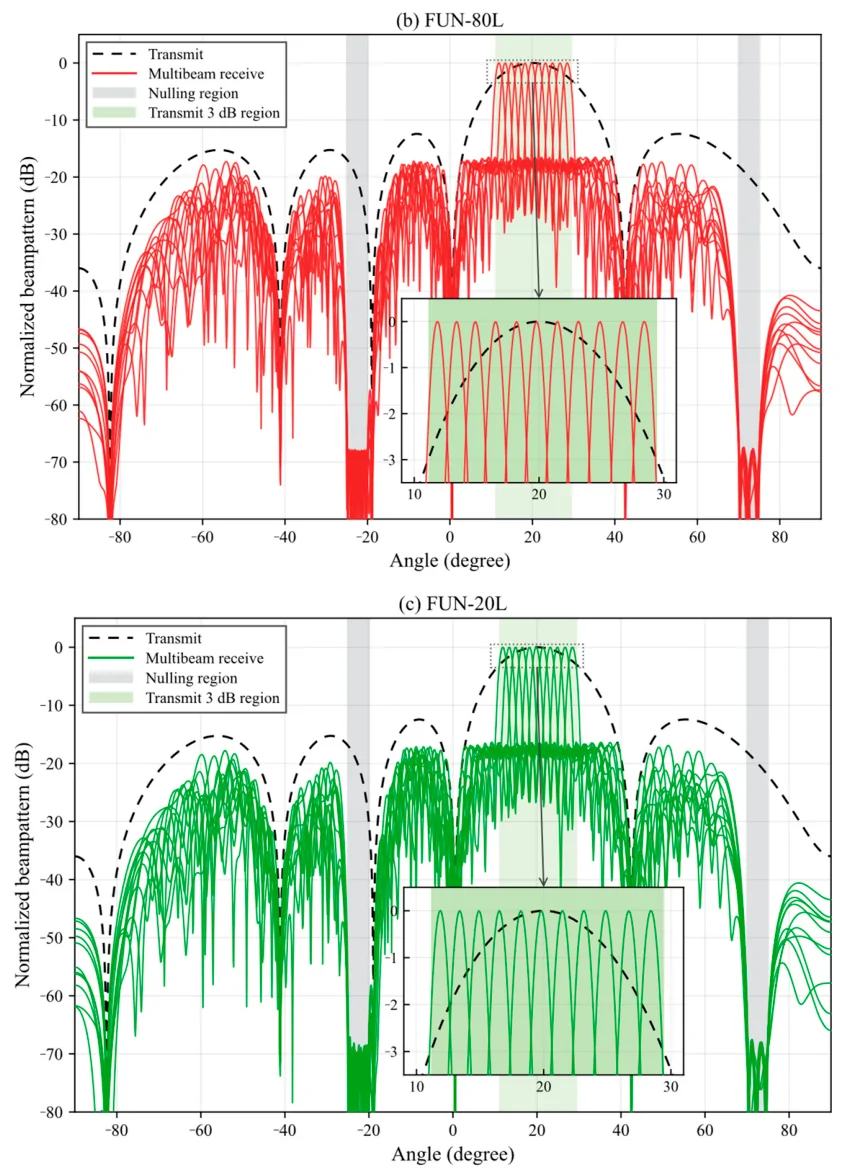
Representative simultaneous focused multi-beam synthesis for the eleven-beam case. (**a**) ADMM, (**b**) FUN-80L, and (**c**) FUN-20L.

**Figure 6:**
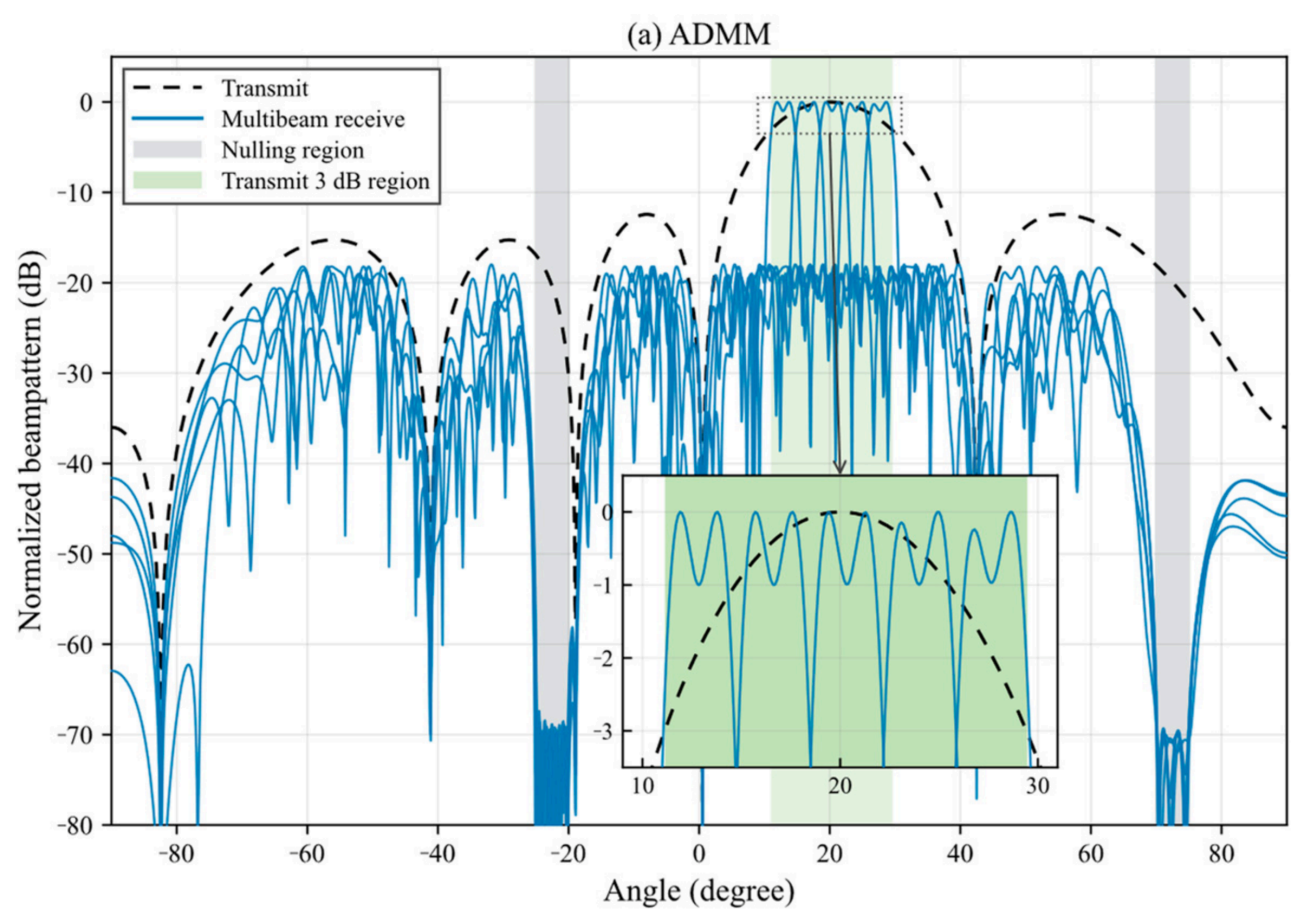

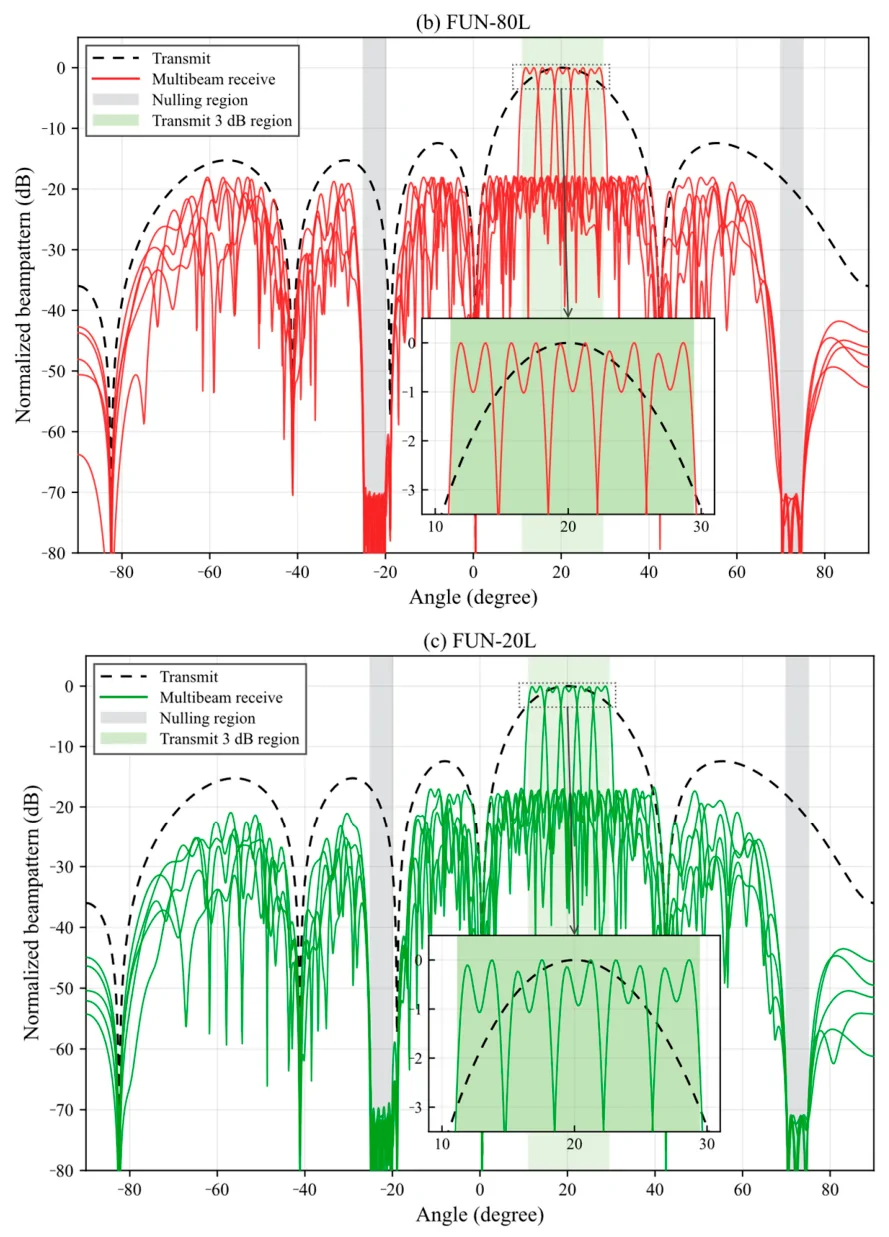
Representative simultaneous narrow flat-top multi-beam synthesis for the five-beam case. (**a**) ADMM, (**b**) FUN-80L, and (**c**) FUN-20L.

Figure 5 and Figure 6 extend the comparison to simultaneous focused and flat-top multi-beam coverage, respectively. The teacher follows the ADMM patterns closely in both beam types. The student retains the coverage geometry but exhibits a finite-depth trade-off in the narrow flat-top setting, where its largest PSL gap is +1.39 dB. The corresponding multi-beam gaps are summarized in Table 3.

Across the six scenarios, the teacher PSL gap lies between −0.29 and +0.49 dB, while the student gap lies between −0.11 and +1.39 dB. Null gaps vary with beam type: the networks are slightly shallower in the focused cases but often deeper in the flat-top cases. These are operating-point differences relative to the fixed-penalty reference, not evidence that the learned model dominates ADMM for every penalty choice.

### 4.3. Convergence and Depth Analysis

**Figure 7:**
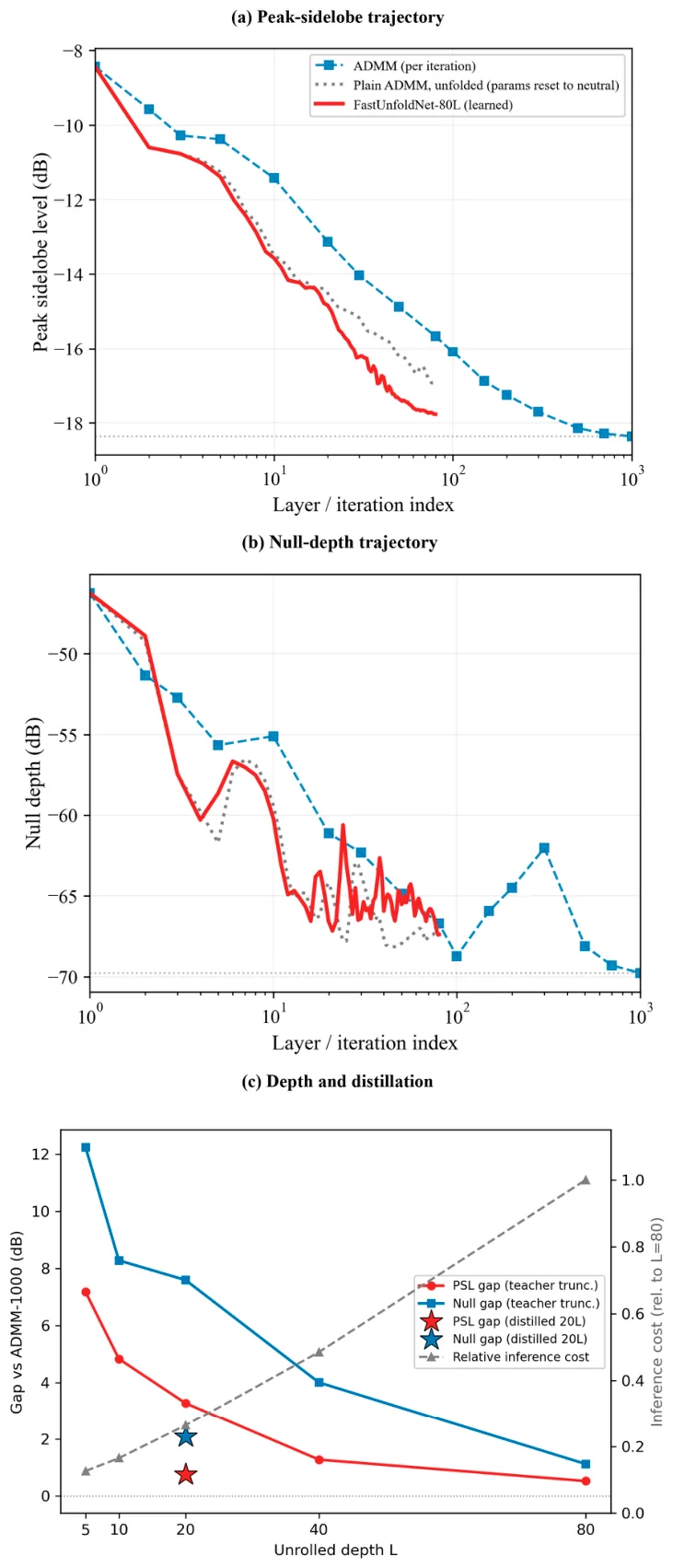
Finite-depth behavior. (**a**) PSL trajectory; (**b**) null-depth trajectory; (**c**) teacher truncation-depth comparison with the 20-layer student.

Figure 7a,b compare the PSL and null-depth progression of ADMM, plain unfolding, and the learned teacher. Within the evaluated update range, the learned teacher achieves lower sidelobe levels while maintaining comparable null suppression relative to the fixed-update baselines. Figure 7c examines the intermediate outputs obtained by truncating the trained 80-layer teacher at 5, 10, 20, 40, and 80 layers. At 20 layers, the separately trained student provides a closer approximation to ADMM than the truncated teacher. This comparison demonstrates the benefit of training a complete short-depth trajectory, although it does not isolate the contribution of teacher guidance without a corresponding student trained without such guidance.

Table 4 extends the analysis to students of 10, 15, 20, 25, and 30 layers trained with the same focused-beam data, four-stage structure, 150-epoch budget, seed 2026, and teacher-based initialization rule. Student checkpoints use the fixed final validation beam-specification loss, excluding teacher guidance. The 80-layer teacher parameters were mapped to 100 and 120 layers, then refined for 40 epochs; the mapped initialization was eligible for selection. Student and teacher scores are defined separately and compared only within their respective families.

The student validation loss decreases from 10 to 25 layers and increases slightly at 30 layers. Thus, 25 layers yields the lowest loss among the tested configurations. The 20-layer student is retained as a more compact configuration requiring fewer sequential updates, at the cost of a moderately higher validation loss than the 25-layer alternative. The deterioration at smaller tested depths does not establish a sharp minimum-depth threshold; identifying such a threshold would require a predefined accuracy criterion.

The 100- and 120-layer teachers achieve lower selection scores than the 80-layer teacher, so the present results do not establish saturation at 80 layers. We therefore do not interpret 80 layers as an optimal or saturation depth; it is retained as the reference teacher used throughout the main experiments and for student initialization and guidance. Likewise, 20 layers is retained as the compact student configuration. The depth study is intended to characterize sensitivity rather than to establish universally optimal depths.

### 4.4. Component Ablation

Table 5 evaluates individual component ablations and a neutral-acceleration configuration without retraining. The comparison separates the finite-depth roles of momentum, relaxation, and stage-wise penalty scheduling within the trained 80-layer teacher.

For the stage-wise-schedule ablation, all stages share the learned first-stage penalty values; in the neutral-acceleration condition, *α* = 1 and *β* = 0 while the learned stage-wise penalties are retained.

Removing momentum produces the largest deterioration in PSL and sidelobe MAE among the individual component ablations, whereas removing the stage-wise penalty schedule increases the null gap from +1.12 to +4.51 dB. Removing relaxation leads to a smaller but consistent degradation across the three reported metrics. When both momentum and relaxation are reset to their neutral values, the PSL gap and sidelobe MAE increase to +1.34 dB and 5.12 dB, respectively, while the learned stage-wise penalties remain active. These results indicate complementary finite-depth roles: momentum and relaxation primarily improve sidelobe fidelity, whereas stage-wise penalty scheduling is particularly important for null suppression.

### 4.5. Computational Efficiency

Efficiency is compared using solver depth, factorization count, parameter count, and asymptotic complexity. The student uses a fixed 20-layer forward pass, whereas ADMM runs until the residual tolerance is met, with 1000 iterations as the maximum. Layer counts and ADMM iterations therefore represent computational budgets rather than equivalent elapsed-time reductions. The hardware-independent comparison is summarized in Table 6.

ADMM requires one reusable factorization, whereas FastUnfoldNet uses one cached factorization per stage (*P* = 4). The student reduces the layer-dependent term from 80 to 20 layers relative to the teacher, without implying a fourfold reduction in end-to-end runtime.

### 4.6. Generalization over Random Focused-Beam Configurations

**Figure 8:**
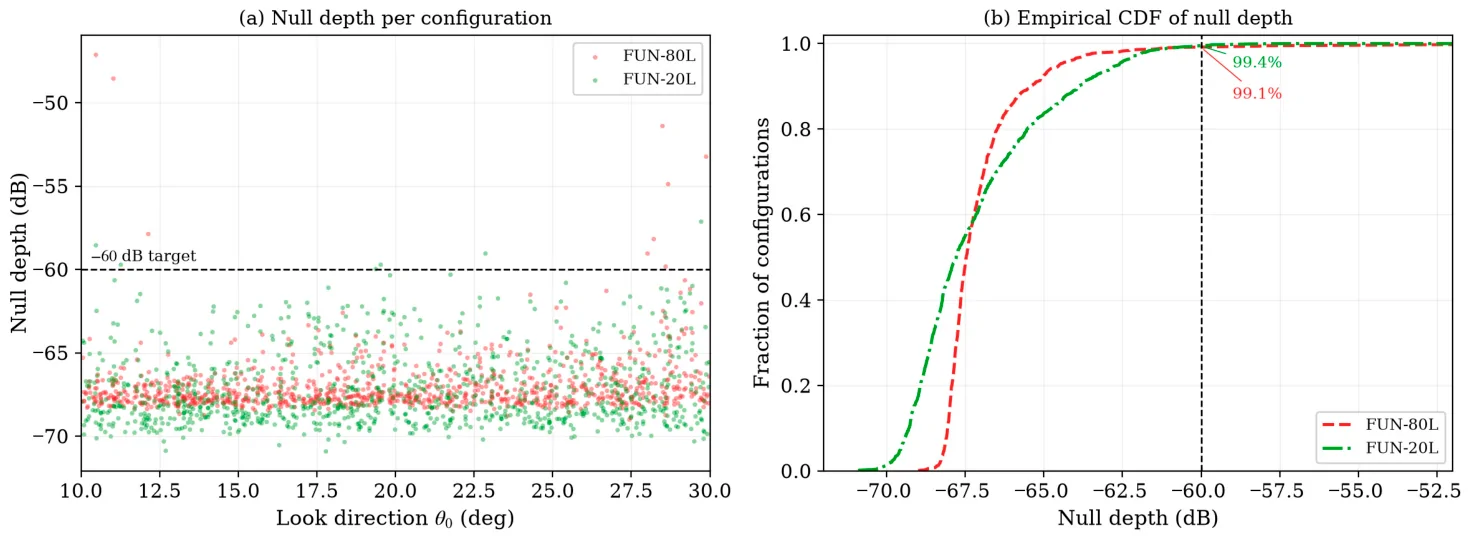
Null-depth results over 1000 random focused-beam configurations. (**a**) Per-configuration null depth; (**b**) empirical CDF.

A 1000-configuration focused-beam sweep evaluates the trained networks over randomly generated look directions and null-region configurations within the tested focused-beam setting. The look direction is sampled from 10° to 30°, and each configuration contains two randomly positioned 5° null regions outside the transmit mainlobe. No network parameter is changed between configurations. Using −60 dB as the reported evaluation threshold, FUN-20L meets the target in 99.4% of cases and FUN-80L in 99.1%. This threshold is less stringent than the −70 dB training target, and the reported success rates should not be interpreted as satisfying the latter target. The resulting null-depth distributions are shown in Figure 8.

This experiment establishes coverage within the tested configuration family only. It does not imply online adaptation or a guarantee under unseen array geometries, angular grids, ripple specifications, calibration errors, or null widths.

### 4.7. Sampled Mainlobe and Null Constraint Violations

We evaluated constraint violations using the final beamforming weights for 1000 focused-beam and 350 flat-top scenarios. ADMM, FUN-80L, and FUN-20L were evaluated on the same scenarios using a common 0.1° angular grid. For each scenario, we calculated the maximum positive deviation from the null-region amplitude upper bound and the mainlobe amplitude lower and upper bounds. A scenario was counted as violating the sampled constraints if any of these deviations exceeded the tolerance. We report tolerances of 10^−6^ and 10^−4^ in the common steering-matrix amplitude scale; these tolerances are not expressed in decibels.

The null-region design bound was −70 dB. The mainlobe bounds were ±0.1 dB for focused beams and ±0.5 or ±1 dB for flat-top beams, depending on the scenario. Table 7 reports the resulting violation rates. ADMM was evaluated with a maximum budget of 1000 iterations.

Violations occur frequently under these tolerances, particularly for the flat-top student. Their magnitude also matters: for the flat-top student, the median, 95th percentile, and maximum mainlobe lower-bound deviations were 0.01181, 0.02162, and 0.07312, respectively, in the common amplitude scale. These deviations cannot all be attributed to numerical roundoff. Although finite-iteration ADMM also exhibits violations, this does not remove the feasibility limitation of the unfolded networks.

The layerwise projections constrain auxiliary responses, whereas feasibility of the final beamforming weights additionally requires consistency between those auxiliary variables and the corresponding weight-generated responses. Such consistency is not guaranteed at finite depth. The results therefore support approximate constrained beam synthesis rather than guaranteed strict feasibility. The −60 dB null-depth success criterion in Section 4.6 is a separate performance measure and does not establish compliance with the −70 dB design bound or the mainlobe bounds. The rates in Table 7 concern the sampled mainlobe and null constraints; they do not certify feasibility over the continuous angular domain.

## 5. Discussion

The experiments support a specific interpretation of FastUnfoldNet: it is a distribution-trained accelerator of a known constrained solver. The inherited projections supply the beamforming geometry, while the learned relaxation, momentum, and stage penalties reshape the finite-depth trajectory. The ablation separates these roles: momentum primarily improves sidelobe behavior, and stage-wise penalties primarily improve null control.

Factorization reuse is central to this interpretation. A fully layer-wise penalty schedule would increase schedule flexibility but require *L* factorizations; fixed-penalty ADMM needs only one. The proposed *P*-stage design occupies the middle point, with *P* factorizations and a learned coarse schedule. Its advantage therefore comes from balancing trajectory flexibility against linear-algebra cost, not from eliminating factorization altogether.

Array geometry is represented explicitly through the steering vectors and the corresponding region matrices. For another subarray layout compatible with the stated signal model, these matrices and the associated stage factorizations must be recomputed, while the form of the ADMM-based updates can be retained. This structural adaptability does not establish that the learned parameters retain their performance on an unseen array without retraining. The present numerical results support performance only for the investigated configuration; evaluation on other array types and assessment of cross-array parameter transfer remain subjects for future work.

Other limitations include the fixed angular grid, separate focused and flat-top models, and comparison primarily with the parent fixed-penalty ADMM solver. The random-scene test remains within the tested configuration family, and robustness to calibration error, quantization, and broader null specifications has not been established. Comparisons with matched-budget learned beamformers and adaptive-penalty optimizers would further clarify the relative contribution of structure and training.

## 6. Conclusions

FastUnfoldNet maps the constrained ADMM beamformer of [7] onto a fixed-depth, physics-structured network that retains the original sidelobe, null, and mainlobe projections and the standard dual update. Stage-wise penalty sharing enables Cholesky factorization reuse, while teacher-response guidance enables the 20-layer student to learn a compact trajectory that is substantially better than direct truncation of the 80-layer teacher at the same depth.

Across the tested beam-synthesis scenarios, the 80-layer teacher remains close to ADMM, while the 20-layer student provides a favorable quality–depth trade-off. The method should therefore be viewed as an interpretable finite-depth accelerator for the tested array and constraint families rather than as a universally superior replacement for iterative optimization. Extending the approach to unified multi-mode models, planar arrays, and hardware-robust training is a natural next step.

## Figures and Tables

**Table 1:** FastUnfoldNet learnable parameters.

Scope	Parameter	Meaning	Count
Global	*ϵ*	Regularization	1
Global	*η*	Null-depth bound	1
Per stage	*ρ_s_*,*ρ_z_*,*ρ_m_*	Region penalties	3*P*
Per layer	*α*	Relaxation	*L*
Per layer	*β*	Heavy-ball momentum	*L*
Teacher	*L* = 80, *P* = 4	Total learnable scalars	174
Student	*L* = 20, *P* = 4	Total learnable scalars	54

**Table 2:** Array, data, training, and reference-solver settings.

Parameter	Value
Array/virtual dimension	*M* = 6, *N* = 6; *D* = 216
Carrier/wavelength	1 GHz/0.3 m
Angular grid	[−90°, 90°], 0.1°
Focused mainlobe	10–30°; ±0.1 dB; transition 1.8°
Flat-top mainlobe	Teacher: 3°, ±0.5 dB; Student: 3–8°, ±0.5/±1.0 dB (≤5°/>5°)
Null regions	2 × 5°; target −70 dB
Training/validation data	Focused: 1000/200; Flat-top: 800/100
Teacher/student	80 L/20 L; 4 stages; 174/54 scalars
Reference solver	ADMM, tolerance 10^(−4)^

**Table 3:** Multi-beam gaps relative to ADMM.

Scenario	Beam Type	Teacher PSL Gap (dB)	Student PSL Gap (dB)	Teacher/Student Null Gap (dB)
S1	11-beam focused	+0.49	+0.77	+0.68/+1.32
S2	7-beam focused	+0.49	+0.96	+1.01/+0.93
S3	5-beam narrow flat-top (±0.5 dB ripple)	+0.13	+1.39	−2.14/−1.88
S4	5-beam narrow flat-top (±0.1 dB ripple)	−0.25	+1.16	−1.05/−2.46
S5	2-beam wide flat-top (±0.5 dB ripple)	+0.33	+0.71	−0.62/+0.81
S6	2-beam wide flat-top (±0.1 dB ripple)	−0.29	−0.11	−0.87/−0.17

**Table 4:** Focused-beam depth sensitivity.

Model	Layers	Student Validation Loss	Teacher Selection Score
Student	10	3.33831	—
Student	15	2.69448	—
Student	20	2.30373	—
Student	25	2.05032	—
Student	30	2.07681	—
Teacher	80	—	0.45008
Teacher	100	—	0.28746
Teacher	120	—	0.17792

**Table 5:** Component ablation of the 80-layer teacher; gaps are relative to ADMM.

Condition	PSL Gap (dB)	Null Gap (dB)	Sidelobe MAE
Full model	+0.52	+1.12	3.07
Without momentum	+1.16	+1.61	4.84
Without relaxation	+0.65	+1.39	3.64
Without stage-wise schedule	+0.38	+4.51	2.78
Neutral acceleration (*α* = 1, *β* = 0)	+1.34	+1.05	5.12

**Table 6:** Hardware-independent computational comparison.

Method	Updates	Factorizations	Learned Scalars	Complexity
ADMM	*K*_ADMM_ ≤ 1000	1	0	*O*(*D*^2^*T* + *D*^3^ + *K*_(ADMM)_(*DT* + *D*^2^))
FUN-80L	80	*P* = 4	174	*O*(*D*^2^*T* + *PD*^3^ + 80(*DT* + *D*^2^))
FUN-20L	20	*P* = 4	54	*O*(*D*^2^*T* + *PD*^3^ + 20(*DT* + *D*^2^))

**Table 7:** Scenario-level violation rates for sampled mainlobe and null constraints. Tolerances are amplitude differences under the common steering-matrix scaling.

Beam Type	Scenarios	Method	Violation Rate 10^−6^ (%)	Violation Rate 10^−4^ (%)
Focused	1000	ADMM	97.90	30.50
Focused	1000	FUN-80L	100	69.30
Focused	1000	FUN-20L	99.00	60.30
Flat-top	350	ADMM	99.43	40.29
Flat-top	350	FUN-80L	99.43	91.71
Flat-top	350	FUN-20L	99.71	99.43

## Data Availability

The synthetic data and code supporting the findings of this study are available from the corresponding author upon reasonable request.

## Appendix A. Structural Properties

**Lemma** **A1** **(well-posed** **cached** **solve).** *Every stage matrix is Hermitian positive-definite, and its condition number admits an explicit upper bound; hence its Cholesky factor exists and the cached triangular solve is backward-stable* [32]*. Indeed, each* $\Phi_{r}^{H}\Phi_{r}≽0$ *and ρ_r_* > 0*, so* $\sum_{r}\rho_{r}\Phi_{r}^{H}\Phi_{r}≽0$ *and* **Q***_s_*≽*ϵ***I**≻0 *is Hermitian; hence λ*_min_(**Q***_s_*) ≥ *ϵ. Its largest eigenvalue obeys* $\lambda_{\max}\left(Q_{s}\right)\leq \epsilon +\sum_{r}\rho_{r}\lambda_{\max}\left(\Phi_{r}^{H}\Phi_{r}\right)=\epsilon +\sum_{r}\rho_{r}∥\Phi_{r}{∥}_{2}^{2}$*, which bounds the condition number.*

**Lemma** **A2** **(parent-update** **consistency).** *With α* = 1 *and β* = 0*, and with the region penalties and global regularization and null-bound parameters fixed at their parent values, the accelerated weight update reduces to the raw solution of Equation (14). For a common input state and the same projection definitions, the resulting primal, projection, and dual operations have the same algebraic form as Algorithm 1. This is an update-form consistency statement and does not imply numerical identity of finite-precision implementations or equality of the complete finite-depth trajectories.*

**Lemma** **A3** **(stability** **region).** *At layer l, the homogeneous part of the recursion (16) is Schur-stable if and only if* |*α*^(*l*)^*β*^(*l*)^| < 1 *and* |1 − *α*^(*l*)^| < 1 + *α*^(*l*)^*β*^(*l*)^*. Indeed, the recursion has characteristic polynomial z*^2^ − (1 − $\alpha^{\left(l\right)}$)*z* + $\alpha^{\left(l\right)}$$\beta^{\left(l\right)}$. *By the Schur–Cohn (Jury) criterion for a monic quadratic z*^2^ + *p*_1_*z* + *p*_2_*, both roots lie inside the unit disk iff* |*p*_2_| < 1 *and* |*p*_1_| < 1 + *p*_2_*; substituting p*_1_ = −(1 − $\alpha^{\left(l\right)}$) *and p*_2_ = $\alpha^{\left(l\right)}$$\beta^{\left(l\right)}$ *gives the condition.*
